# Magnesium Sulfate-Rich Natural Mineral Waters in the Treatment of Functional Constipation–A Review

**DOI:** 10.3390/nu12072052

**Published:** 2020-07-10

**Authors:** Christophe Dupont, Guillaume Hébert

**Affiliations:** 1 Pediatric Department, Necker Enfants-Malades Hospital, Paris-Descartes University, 75015 Paris, France; 2 Clinique Marcel Sembat, 92100 Boulogne Billancourt, France; 3 SC Partners, 75014 Paris, France; guillaume.hebert@sc-partners.eu

**Keywords:** bowel movement, functional constipation, magnesium, mechanism of action, natural mineral water, natural treatment, osmotic effect, sulfate

## Abstract

Functional constipation (FC) is a chronic constipation for which no physiological, anatomical or iatrogenic origin can be evidenced. This condition has a high impact on a patient’s quality of life and healthcare costs. Since FC is frequently associated with low physical activity and a diet low in fiber and/or water, first-line recommendations focus on sufficient activity, and sufficient fiber and water intake. In case of inefficacy of these measures, numerous drug treatments are available, either over the counter or on prescription. Magnesium sulfate has a long history in the treatment of FC, and magnesium sulfate-rich mineral waters have been used for centuries for their laxative properties. The laxative effect of magnesium and sulfate has since been widely demonstrated. Nevertheless, it appears that no clinical studies aiming at demonstrating their efficacy in FC had been conducted before the 21st century. In this paper, we reviewed the clinical data reporting the efficacy of magnesium sulfate-rich natural mineral waters. In view of their reported efficacy and safety, magnesium sulfate-rich natural mineral waters may represent a natural treatment for FC.

## 1. Functional Constipation

Functional constipation (FC) is a common condition with a worldwide lifetime prevalence of approximately 14% [1,2,3,4,5]. FC is more frequent in females than in males, with a 2/1 sex ratio. It is also associated with increasing age, lower socioeconomic status, low physical activity, low fiber intake and low water intake, but also with low magnesium intake [6,7]. It is associated with a high impact on patients’ quality of life, and high healthcare and other indirect costs [8,9,10].

From a general point of view, constipation can be defined simply as the too rare output of too hard stools (i.e., type 1 or 2 on the Bristol Scale of Fecal Consistency) [11]. More precisely, constipation corresponds to difficult, infrequent, or incomplete defecation. However, these definitions cover a wide range of very different conditions with very different etiologies and/or pathophysiologies. FC corresponds to constipation for which no major anatomical, physiological or iatrogenic origin can be identified. For instance, it excludes opioid-induced constipation, Hirschsprung’s disease, anorectal malformations, chronic intestinal pseudo-obstructions, encephalopathies, myopathies, spinal cord lesions and hypothyroidism, as well as metabolic disorders like hypokalemia, hypocalcemia and hypomagnesemia.

In their last update, the Rome IV criteria diagnose FC according to the symptoms presented in Table 1 [12]. These symptoms should have appeared at least six months before and be observed during the last three months. In addition, patients should not meet the criteria for Irritable Bowel Syndrome with Constipation (IBS-C), another functional bowel disorder. Abdominal pain or bloating can be present but must not predominate.

## 2. Current Treatments

The overall principle for the treatment of FC is a combination between stool softening and decreased intestinal transit time.

### 2.1. First-Line Recommendations

In FC, no drugs are currently recommended as a first-line treatment. First-line recommendations only include modifications of daily practices. As a consequence, therapeutic education should be provided to the patient concerning lifestyle changes and nutritional-hygienic measures [3,5,8,9,10]. These include increased physical activity to improve the colonic transit and increase the number and weight of stools, progressive increase in fibers intake up to 15–40 g per day, and sufficient water intake (i.e., 1.5–2.0 L per day).

Lifestyle changes and nutritional-hygienic measures should improve the symptoms of FC, but they may reveal insufficient. Therefore, different drug treatments are available, some of them being of natural origin.

### 2.2. Drug Treatments

Different drug treatments are available (for review, see reference [13]) (Table 2). They all aim to increase the weight and/or volume of the stools, which increases intestinal contractions. Their major side effects can be increased flatulence, abdominal distension and abdominal pain, mainly observed for bulk laxatives and saccharides. Please refer to the respective summary of product characteristics of each individual treatment for detailed information concerning its indications, contraindications, and undesirable effects.

#### 2.2.1. Bulk Laxatives

Bulk laxatives act by increasing the volume of stools using their high hydrophilic power. They are extracts of algae, gums or greases, and include psyllium, calcium polycarbophil, bran and methylcellulose. Increased stool volume induces intestinal peristaltic reflex and allows a better sphincter reflex.

#### 2.2.2. Lubricants and Emollients

Lubricants are nontoxic mineral oils (e.g., paraffin oils) that are not absorbed by the intestinal epithelium. They lubricate the intestinal wall and improve the passage of stools. Emollients are surfactants (e.g., docusate) that increase the stool content in water and lipids. Emollients and lubricants soften stools and decrease intestinal water reabsorption.

#### 2.2.3. Stimulant Laxatives

Stimulant laxatives directly act by stimulating intestinal motility and, to a lower proportion, increase water secretion into the intestinal lumen. These laxatives actively increase the secretion of water by the intestinal mucosa into the intestinal lumen. They include bisacodyl, senna and sodium picosulfate.

#### 2.2.4. Osmotic Laxatives

As their denomination clearly indicates, osmotic laxatives exert an osmotic effect that increases water content of the intestine. This action is mediated through a passively increased water secretion by the intestinal epithelium and water retention in the intestinal lumen.

##### Saccharides

Saccharides are fermentable sugars that are not digested in the small intestine and act following their degradation by the colonic microbiota. They include lactitol, lactulose, mannitol, pentaerythritol and sorbitol. Saccharides are transformed into short chain fatty acids in the colon and exert an osmotic effect. Subsequently, short chain fatty acids also act as prebiotics that accelerate the development of gut microbiota, which is supposed to soften and increase the weight of stools.

##### Polyethylene Glycols (PEG)

PEGs are long linear polymers, named macrogol, of which molecular weight is most frequently 3350, 4000 or 6000 g/mol. PEGs are often associated with electrolytes. They do not cross the intestinal barrier and they retain water molecules through hydrogen bonds, thereby softening stools and increasing their volume.

##### Magnesium Oxide

Magnesium-containing drugs, also called “milk of magnesia”, are prescribed commonly for the treatment of two different gastrointestinal conditions, depending on the dosage. Magnesium oxide (MgO, powder) and magnesium hydroxide (Mg(OH)_2_, in solution) are the same drug; magnesium oxide transforms into magnesium hydroxide in the presence of water: MgO + H_2_O –> Mg(OH)_2_.

At low doses (<2 g/day), magnesium hydroxide is an active compound of antacid drugs (e.g., Maalox). In this indication, its effect is purely chemical. Stomach protons react with hydroxide and produce water: Mg(OH)_2_ + 2H^+^ –> Mg^2+^ + 2H_2_O.

At higher doses (≥2 g/day), magnesium hydroxide is used as an osmotic laxative, either per os or in enema preparations. The part of Mg(OH)_2_ that is not transformed into Mg^2+^ in the stomach is transformed into magnesium carbonate (MgCO_3_) in the intestine. Of note, because of the high doses of magnesium in magnesium oxide-based laxatives, a risk of hypermagnesemia has been associated with their use, in particular in the case of laxative abuse [14,15,16,17,18,19]. Some cases of death have been reported [20,21].

### 2.3. Probiotics

The World Health Organization and Food and Agriculture Organization define probiotics as follows: “live microorganisms which when administered in adequate amounts confer a health benefit on the host”. In FC, the use of probiotics aims at improving composition of the gut microbiota. Based on the findings demonstrating altered gut microbiota in patients with FC and the fact that saccharides also act through a prebiotic effect, the efficacy of probiotics on FC has been investigated [22,23,24]. Despite controversial results, it appears that probiotics could be useful in the long-term treatment of FC. The efficacy of probiotics seems to highly depend on the microbial strain and is observed essentially when at least two different strains are used. The exact mechanism of action of probiotics still remains unclear, but it most probably involves stool composition (e.g., production of short fatty acids), stool weight and intestinal peristalsis [25,26,27,28].

## 3. Magnesium Sulfate-Rich Natural Mineral Waters

Magnesium sulfate, also named Epsom salt since the 17th century, has a long history in the treatment of constipation. As a medicine, magnesium sulfate was patented by John Callen in 1818 [29]. “Fluid Magnesia” was used for the first time in 1829 by Sir James Murray to treat a patient with stomach pain, apparently with great success. Since the early 20th century, the pharmacological effect of magnesium sulfate on constipation has been studied [30].

However, the clinical effect of magnesium sulfate-rich natural mineral waters has been evaluated only recently. Possibly because of the evidenced laxative effect of magnesium sulfate, the intrinsic laxative effect of these natural mineral waters was not questioned. In addition, natural mineral waters cannot be modified, as they would not be natural anymore. To our knowledge, only three magnesium sulfate-rich natural mineral waters have been clinically studied in FC: Hépar in 1999, 2014 and 2019 [31,32,33], Ensinger Schiller Quelle in 2016 [34] and Donat Mg in 2017 [35] (Table 3). The mineral content of these waters is presented in Table 4.

### 3.1. Hépar

Hépar (Nestlé Waters, France) is a magnesium sulfate-rich natural mineral water marketed in France since 1930. This spring was discovered in 1873 and first referenced in 1875 by the French Academy of Medicine, which reported that this “salted spring” was “known […] as a purgative spring” [36]. “The salted spring” was declared of “public utility” in 1903 and renamed Hépar in 1919 [37]. In France, people frequently consume Hépar to alleviate their symptoms of constipation. Three clinical trials were conducted to evaluate the efficacy of Hépar in FC [31,32,33].

The first study of the effect of Hépar in the treatment of FC was successfully conducted in infants [31]. This randomized, double blinded, placebo-controlled study included 60 infants with FC (mean age 1.9 ± 0.9 months). Unfortunately, that study has not been published in an international peer-reviewed journal and will not be discussed further in this paper.

The second clinical study we conducted to evaluate the laxative effect of Hépar was published in 2014 [32]. This randomized, double blind, placebo-controlled study was conducted in 244 women aged 18 to 60 years and diagnosed FC according to the Rome III criteria. Constipation was mild in 16.8% of the patients, moderate in 64.8% and severe in 18.4%. To avoid any constipation due to insufficient water intake, patients were included after an eight to nine day washout period during which they should drink 1.5 L/day of low-mineral spring water (400 vs. 2513 mg/L for Hépar). Patients were randomly allocated to three treatment groups, which all should drink at least 1.5 L water per day. These groups were: (1) 1.5 L low mineral water (placebo group), (2) 1 L low mineral water plus 0.5 L Hépar and (3) 0.5 L low mineral water plus 1 L Hépar. Patients could use PEG in case of too intense abdominal pain. Because the major goals to reach in the treatment of FC are improved intestinal transit time and stool softening, response to the treatment was defined as a composite score specially designed for the purpose of this study, based on two of the Rome III criteria. Responder should have four or more stools/week or an increase of two or more stools per week, but also less than 25% lumpy or hard stools.

A significant effect of the intake of 1 L Hépar per day was observed during the second week of treatment. The proportion of responders was 37.5% with 1 L Hépar vs. 21.1% in the placebo group (*p* = 0.013). This difference was also observed at the end of the study (week 4, 39.0% vs. 24.3%, respectively; *p* = 0.028). The use of 1 L/day Hépar was associated with a decreased number of lumpy or hard stools, without any increase in the number of fluffy or liquid stools. In addition, patients using 1 L/day Hépar dramatically decreased their use of rescue laxatives. At week 4, 19.7% patients of the placebo group used PEG versus 2.8% in the Hépar group (*p* = 0.001). Data showed that the proportion of responders to the treatment was correlated to the weekly Mg_2_^+^ and SO_4_^2-^ osmolarity of the treatment. Study discontinuation was reported for 9/82 patients in the 1 L Hépar group vs. 8/77 patients in the placebo group. Compliance to the treatment was 101.6% ± 13.6% of the theoretical consumption in the 1 L Hépar group. No serious adverse events (AEs) were reported. Two cases of diarrhea related to the treatment were reported in the Hépar group vs. two patients in the placebo group.

The third clinical study of the efficacy of Hépar in FC focused on the analysis of time to treatment response [33]. Inclusion and efficacy criteria were the same as in the first study. The efficacy of this magnesium sulfate-rich mineral water was confirmed by this study including 226 patients, 80.8% with mild constipation and 19.2% with moderate constipation. After two weeks, the proportion of responders was 50% in the patients using Hépar versus 29% in the placebo group (*p* = 0.001). In addition, this study showed that response to the treatment was reached in a mean of 6.4 days following treatment initiation.

Study discontinuation was reported for 3/113 patients in the Hépar group and 2/113 in the placebo group. Compliance to the treatment was 93.4% ± 10.8% of the theoretical consumption in the 1 L Hépar group. One case of abdominal bloating and one case of meteorism were related to the treatment, in the Hépar group.

### 3.2. Ensinger Schiller Quelle

Ensinger Schiller Quelle (Ensinger Mineral-Heilquellen, Germany) is a magnesium sulfate-rich natural mineral water with added carbon dioxide. Apart from the fact that this mineral water is rendered sparkling by the addition of 2650 mg/L carbon dioxide, its total mineralization and mineral content are very similar to Hépar (Table 3). The efficacy of this water in FC was evaluated in a randomized, double blind, placebo-controlled clinical study published in 2016 [34]. This study included 100 patients (85% females) aged 18 to 64 years diagnosed with mild or moderate FC according to the Rome III criteria (i.e., 2–4 bowel movements/week). Preceding inclusion, participants had a one-week run-in period, during which they should have two to four complete bowel movements and drink 1 L water per day. Following inclusion, patients had to drink 0.25 L water four times daily at precise moments, during a six-week period of time. Total mineralization was 2666 mg/L for the test water. Placebo was tap water (108 mg/L minerals) added with carbon dioxide. The use of rescue medication was not allowed during the study. Evaluations were performed at three and six weeks following inclusion. Endpoints were based on the number of bowel movements.

A statistically significant effect was observed at three weeks of treatment. The number of bowel movements was increased in the treatment group (2.02 ± 2.22 movements/week) as compared to the placebo group (0.88 ± 1.67, *p* = 0.005). At week 3, the treatment group also showed improved stool consistency (Bristol scale) as compared to the placebo group (3.1 vs. 2.7, respectively; *p* = 0.044). However, this study did not show improvement in stool frequency or consistency at week 6.

No study discontinuation was reported for any of the 50 patients of the 1 L ESQ placebo group. Compliance to the treatment was 97% ± 5.4% of the theoretical consumption in the 1 L ESQ group. No serious AEs were reported. Minor meteorism related to the treatment was reported for one patient in the 1 L ESQ group vs. one case of diarrhea in the placebo group.

### 3.3. Donat Mg

Donat Mg (Droga Kolinska, d.d, Slovenia) is a magnesium sulfate-rich natural mineral water produced in Slovenia. Donat MG has very high mineral contents (13,000 mg/L) as compared to Hépar (2513 mg/L) or Ensinger Schiller Quelle (2666 mg/L).

The randomized, double blind, placebo-controlled clinical study of the effect of Donat Mg in FC was published in 2017 [35]. It included a total of 106 patients (84% females) aged 18 to 70 years and diagnosed with mild or moderate FC according to the Rome III criteria (i.e., 2–4 bowel movements/week). The use of rescue medication was allowed. Participants were included following a 10 day run-in period, during which they should have 2–4 bowel movements weekly and drink ≥ 0.3 L water daily. Two arms were constituted, 30 patients were allocated to 0.3 L water per day (treatment or placebo) and 75 patients were allocated to 0.5 L/day (treatment or placebo). Placebo was a low-mineral sparkling water (500–600 mg/L minerals plus 3500 mg/L CO_2_). The 0.3 L arm was prematurely stopped, and results have not been presented. Endpoints were based on the number of bowel movements.

Results showed an overall improvement in the number of spontaneous bowel movements in the treatment group as compared to the placebo group at three weeks (6.14 ± 3.32 vs. 4.45 ± 2.09, respectively; *p* = 0.006) and six weeks of treatment (6.62 ± 3.20 vs. 4.47 ± 2.20, respectively; *p* = 0.001). Patients in the treatment group reported more frequently softer stools than in the placebo group at weeks 3 and 6 (*p* < 0.001).

Study discontinuation was reported for 1/38 patients in the Donat Mg group vs. 0/38 patients in the placebo group. Compliance to the treatment was 102% ± 8.6% in the Donat Mg group. Three AEs were related or probably related to the Donat Mg treatment.

### 3.4. Mechanisms of Action

Studies evidenced that magnesium and sulfate both individually exert a laxative action. This is mainly mediated by an osmotic effect due to their incomplete absorption in the gastrointestinal tract. Concerning sulfates, the use of water with high sulfate contents has been shown to be laxative [38,39]. Intestinal sulfate absorption was shown to be limited [40]. This is also supported by the rapid efficacy of oral sulfates in preparations for colonoscopy [41]. Under physiological conditions, magnesium absorption ranges between 30% and 50% of the dose ingested. It can vary from 20% to 80% under extreme conditions (e.g., hypermagnesemia or hypomagnesemia, respectively) and seems to be dose-dependent [42]. Intestinal magnesium absorption occurs between 1 and 6 h following oral intake (for review, see reference [43]). 

**Figure 1:**
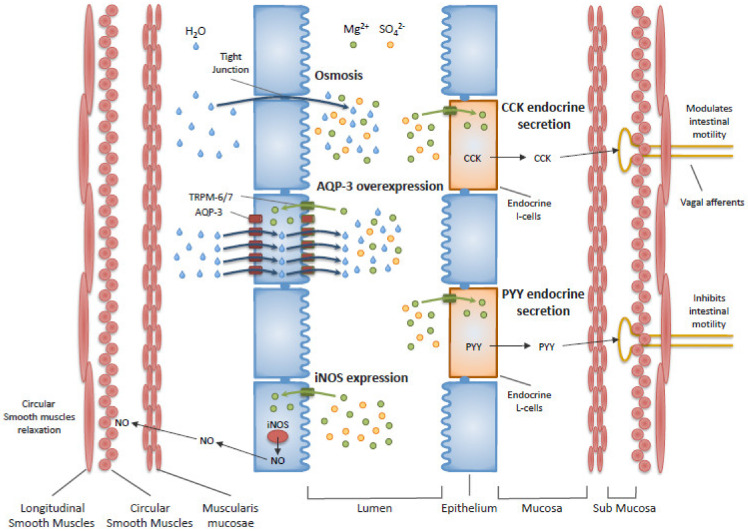
Schematic representation of the five mechanisms of action currently proposed for magnesium sulfate in functional constipation: Osmosis, AQP-3 overexpression, iNOS expression, CCK endocrine secretion and PYY endocrine secretion. AQP-3—Aquaporin-3; CCK—Cholecystokinin; iNOS—inducible Nitric Oxide Synthase; PYY—Peptide YY; TRPM—Transient Receptor Potential Melastatin.

In addition to the osmotic effect of magnesium sulfate, the presence of another mechanism of action was first proposed in 1939 [44]. This actually concerns magnesium, for which the involvement of cholecystokinin (CCK) was suggested in 1973 [45], nitric oxide synthase (NOS) in 1994 [46] and aquaporin-3 (AQP-3) in 2011 [47,48]. Since then, other studies have showed a role of CCK and peptide YY (PYY) endocrine secretions [49], increased expression of inducible NOS and antimicrobial action of magnesium [23,46,50,51]. These mechanisms require an intracellular transport of magnesium, which is allowed by the membrane transient receptor potential melastatin (TRPM) types 6 and 7 [43]. Figure 1 presents these mechanisms of action currently proposed for the effect of magnesium sulfate on stool softening and gut motility. These mechanisms of action are still debated and will not be further detailed in this paper.

Regarding sulfates, we did not find any study reporting a mechanism another than osmotic, which appears to be the principal mechanism of action in FC. Nonetheless, it may be interesting to investigate the impact of dietary sulfates on gut microbiota, especially regarding sulfate-reducing bacteria. Although putative, such a prebiotic mode of action would be in accordance with the time to treatment response (i.e., six days) we observed using Hépar [33].

### 3.5. Overview

Four studies published in international peer-reviewed journals were conducted in adults and analyzed the effect of three different magnesium sulfate-rich natural mineral waters in FC. Overall, these randomized, double blinded, placebo-controlled studies were well-conducted and had similar designs: (1) FC diagnosed according to the Rome III criteria, (2) a 7 to 10 day run-in period before inclusion, (3) use of a low-mineral water as the placebo, (4) study durations between four and six weeks, (5) compliance to the treatment very close to 100% in all studies, and (6) excellent safety results.

Nonetheless, differences can be observed in the design of these studies. All the studies had a run-in period, during which consumption of a minimum amount of water was required. These amounts corresponded to the amount to be consumed during the treatment period (i.e., 0.3 L for Donat Mg, 1 L for ESQ and 1.5 L for Hépar), but they largely differed between studies. In addition, in the ESQ and Donat Mg studies, there was no requirement concerning the type of water (still, sparkling or tea) to be used in this period. As underlined by Naumann et al. [34], because the placebo was sparkling water in these two studies, this modification between the run-in and the treatment period might partly explain the increased number of bowel movements observed in their respective placebo groups [52,53]. Another bias may be attributed to a “study participation” effect according to which participants probably paid more attention to their dietary habits and/or total consumption of water, further increasing FC improvement in the placebo groups. Because the characteristic taste of magnesium sulfate-rich mineral waters is easily recognizable, a double effect may also be hypothesized, which could hardly be quantified. If a placebo effect can easily be imagined in the treatment group [54], at least part of the patients in the placebo groups may have experienced a nocebo effect when understanding that they had not been allocated to the treatment group. Furthermore, a nocebo effect can also be envisaged in patients of the treatment group who had already tried one of these mineral waters but visited their physician because it had failed to improve their symptoms. Nevertheless, although unbiased studies are always expected, the major biases observed in these studies seem in favor of the placebo group. By improving symptoms in the placebo group, these biases most probably decreased the power of the studies and avoided exact quantification of the intrinsic efficacy of magnesium sulfate-rich mineral waters in FC.

An important fact to consider is that the studies presented in this review could not be designed on the basis of previous clinical results, which would have helped a priori estimation of the effects to expect. This is probably the major reason why the primary objective was never met, except in our second study on Hépar. The design of further clinical studies should benefit from the data reported by those studies, including response rates in the placebo group, time to treatment response, quantities of magnesium sulfate, and primary endpoint to be used. In addition, to our knowledge, no studies have been conducted to compare the efficacy, safety or observance of magnesium sulfate-rich natural mineral waters to that of any of the currently used drug treatments. As a consequence, the results presented in this review can only report to the intrinsic efficacy and safety of these waters, as compared to placebo.

Despite these methodological difficulties, all the reported studies demonstrated efficacy of magnesium sulfate-rich mineral waters in the treatment of FC. The consumption of magnesium sulfate-rich water was associated with a significant increase in the number of bowel movements (+1–2 bowel movements/week) and improvement in stool consistency, as compared to the placebo. Depending on the study design, significance was reached during the second or the third week following treatment initiation. In the second study on Hépar, we showed that participants who responded to the treatment had treatment response in a mean of 6.4 days following inclusion. Apart from the ESQ study (six weeks) [34], efficacy was observed until the end of the study in the two other studies that performed measurements over time (i.e., four weeks for Hépar and six weeks for Donat Mg) [32,35].

Meta-analysis could be useful to compare the intrinsic efficacy of the three waters studied. Based on available data, it seems that Donat Mg has a greater effect than Hépar or ESQ. Considering the osmotic mode of action of magnesium and sulfate, it may easily be hypothesized that differences were due to differences in the respective magnesium and sulfate contents of these waters. This is supported by numerous studies (please refer to the Mechanism of action section) and by our study, showing that response to the treatment was correlated to the amount of magnesium and sulfate consumed [32]. Donat Mg has extremely high mineral contents (Table 4). We did not find any evidence that minerals other than magnesium or sulfate exert a sufficiently high effect to improve FC. Because the osmotic effect does not depend on the weight of minerals but on their number, Table 5 presents the number of magnesium and sulfate particles consumed daily in each of the four studies (in mMol). The two groups, for which no significant effect on FC symptoms was reported, had the lower content in magnesium or sulfate (10.5 and 18.8 mMol/day). The groups showing efficacy in FC were 1 L ESQ (20.4 mMol/day), 1 L Hépar (20.9 mMol/day) and 0.5 L Donat Mg (31.2 mMol/day). According to these data and considering the mechanisms of action of magnesium sulfate, one may hypothesize that the effect of 0.5 L/day Donat Mg could be reached with ESQ or Hépar at the quantity of 1.5 L/day. This is further supported by the excellent safety results observed in each study.

## 4. Conclusions

Magnesium sulfate-rich natural mineral waters have been used for centuries in the treatment of numerous gastrointestinal conditions. Three of these waters have been clinically evaluated for the treatment of FC in four randomized, double blind, placebo-controlled studies. Each of these studies evidenced a laxative effect of magnesium sulfate-rich natural mineral waters, in association with a very good safety profile. They all showed improved stool consistency and number of bowel movements. The current data relating to magnesium sulfate-rich natural mineral waters clearly indicate that they may represent a natural treatment for adult patients with FC. Most probably because of their osmotic mechanism of action, efficacy was more noticeable with higher total concentration of magnesium and sulfate. In view of the reported results, the authors suggest that at least 20 mMol of magnesium sulfate should be consumed daily during at least one week in the treatment of FC, which corresponds to 0.5 L Donat Mg, 1 L ESQ or 1 L Hépar.

Clinical investigation of the laxative effect of magnesium sulfate-rich natural mineral waters is a very recent field of research. Additional studies may be of interest to compare their efficacy and safety to that of other chronic treatments for FC. Observational studies could provide their real-life efficiency and safety, including in the long-term, as well as data regarding the population using these waters. Of note, particular attention should be paid to the entire composition of mineral waters. In view of the current recommendations from the WHO concerning sodium consumption (i.e., ≤2000 mg/day), some waters may be inappropriate for patients at risk of hypertension or cardiovascular disease [55].

## Figures and Tables

**Table 1:** Rome IV criteria for the diagnosis of functional constipation [12].

Criteria
**1. Must include 2 or more of the following:**
a. Straining during more than 25% of defecations
b. Lumpy or hard stools (Bristol Scale 1–2) more than 25% of defecations
c. Sensation of incomplete evacuation more than 25% of defecations
d. Sensation of anorectal obstruction/blockage more than 25% of defecations
e. Manual maneuvers to facilitate more than 25% of defecations (e.g., digital evacuation, support of the pelvic floor)
f. Fewer than 3 spontaneous bowel movements per week
**2. Loose stools are rarely present without the use of laxatives**
**3. Insufficient criteria for irritable bowel syndrome**

Symptom onset at least six months before and observed during the last three months.

**Table 2:** Summary of the current treatments for functional constipation.

	Active Compound	Mechanisms of Action	Major Undesirable Effects
**First line recommendations**	/	- Lifestyle changes and nutritional-hygienic measures	/
**Drug treatments**			
Bulk laxatives	e.g., psyllium, calcium polycarbophil, bran, methylcellulose	- Increase stool volume due to their high hydrophilic power	- Flatulence and abdominal distension
Lubricants	Mineral oils (e.g., paraffin oils)	- Lubricate the intestinal wall	- Anal irritation, lipoid pneumonia if inhaled
Emollients	Surfactants (e.g., docusate)	- Increase the stool content in water and lipids	- Nausea, abdominal cramps
Stimulant laxatives	e.g., bisacodyl, senna and sodium picosulfate	- Stimulate intestinal motility and increase water secretion	- Abdominal discomfort, nausea, cramps
Osmotic laxatives			
Saccharides	Non-digested fermentable sugars (e.g., lactitol, lactulose, mannitol, pentaerythritol and sorbitol)	- Transformed into short chain fatty acids exerting an osmotic effect and acting as prebiotics	- Flatulence, abdominal pain, nausea, vomiting, bloating
Polyethylene glycols	3350, 4000 or 6000 g/mol PEGs	- Do not cross the intestinal barrier and retain water in the intestine	- Nausea, abdominal distension, cramps
Magnesium oxide	MgO	- Low intestinal absorption, osmotic effect	- Hypermagnesemia
Probiotics	e.g., lactobacilli, bifidobacteria	- Probably modification of the gut microbiota and production of short chain fatty acids	Not reported

**Table 3:** Studies of the effect of magnesium sulfate-rich mineral waters in functional constipation.

Author, Year (Ref)	Design	Treatment	Comparator	Primary Endpoint	Outcome
Dupont et al. 2014 [32]	RCT, 244 participants (100% females), Rome III criteria	Hépar 0.5 or 1 L/day	Low mineral natural water (400 mg/L)	Response to the treatment: ≥4 stools/week or an increase of ≥2 stools per week AND <25% lumpy or hard stools	- Increased response rate with 1 L/day Hépar vs. placebo: +16.4% at week 2 (*p* = 0.013) +14.7% at week 4 (*p* = 0.028)
Naumann et al. 2016 [34]	RCT, 100 participants (85% female), Rome III criteria with 2–4 bowel movements/week and ≥ 1 L/day water	Ensinger Schiller Quelle 4 × 0.25 L/day	Tap water (108 mg/L) + 2650 mg/L CO_2_	Difference in stool frequency	- Increased weekly number of stools at 3 weeks vs. placebo (4.80 vs. 3.82, *p* = 0.036)
Bothe et al. 2017 [35]	RCT, 75 participants (84% females), Rome III criteria with 2–4 bowel movements/week and ≥ 0.3 L/day water	Donat MG 0.5 L/day	Low mineral natural water (<1000 mg/L) + 3500 mg/L CO_2_	Difference in complete spontaneous bowel movement	- Increased weekly bowel movements vs. placebo: at 3 weeks 6.14 vs. 4.45, *p* = 0.006 at 6 weeks 6.62 vs. 4.47, *p* = 0.001.
Dupont et al. 2019 [33]	RCT, 226 participants (100% females), Rome III criteria	Hépar 1 L/day	Low mineral natural water (400 mg/L)	Response to the treatment: ≥4 stools/week or an increase of ≥2 stools per week AND <25% lumpy or hard stools	- Increased response rate vs. placebo at 2 weeks: +21.1% responders (*p* = 0.001) Response reached in a mean of 6.4 ± 0.6 days (*p* = 0.013 vs. placebo)

RCT: Randomized, double-blind, placebo-controlled trial.

**Table 4:** Mineral content (mg/L) of the studied natural mineral water.

	Hépar [32,33]	ESQ [34]	Donat Mg [35]
**Minerals**	**2513**	**2666**	**13000**
Magnesium (Mg^2+^)	119	105	1000
Sulfate (SO_4_^2-^)	1530	1535	2000
Calcium (Ca^2+^)	549	573	370
Carbonates (HCO^3-^)	383.7	347	7600
Sodium (Na^+^)	14.2	28.9	1600
Potassium (K^+^)	4.1	7.34	/
Nitrates (NO^3-^)	4.3	2.7	/
Chloride (Cl^-^)	18.8	31.4	/
**Carbon dioxide (CO_2_)**	/	**2650**	**3800**

ESQ: Ensinger Schiller Quelle.

**Table 5:** Magnesium and sulfate consumed daily by the patients (mMol).

	Molar Mass	0.5 L Hépar [32]	0.3 L Donat Mg [35]	1 L ESQ [34]	1 L Hépar [32,33]	0.5 L Donat Mg [35]
Mg^2+^	24 g/Mol	2.48	12.50	4.38	4.96	20.83
SO_4_^2−^	96 g/Mol	7.97	6.25	15.99	15.94	10.42
Total		10.45	18.75	20.37	20.90	31.25
Efficacy *		-	-	+	+	+

ESQ: Ensinger Schiller Quelle; * Improved the weekly number of bowel movement and/or stool consistency.
